# High-risk gastrointestinal stromal tumour (GIST) and synovial sarcoma display similar angiogenic profiles: a nude mice xenograft study

**DOI:** 10.3332/ecancer.2017.726

**Published:** 2017-03-09

**Authors:** Francisco Giner, Isidro Machado, Jose Antonio Lopez-Guerrero, Empar Mayordomo-Aranda, Antonio Llombart-Bosch

**Affiliations:** 1 Department of Pathology, Universitat de València Estudi General (UVEG), València 46010, Spain; 2 Laboratory of Molecular Biology, Fundación Instituto Valenciano de Oncología (FIVO), Valencia 46009, Spain; 3 Department of Pathology, Fundación Instituto Valenciano de Oncología (FIVO), Valencia 46009, Spain.

**Keywords:** GIST, synovial sarcoma, angiogenesis, nude mice xenograft, chemokines

## Abstract

**Background:**

Gastrointestinal stromal tumour (GIST) is the most common primary mesenchymal tumour of the gastrointestinal tract. Spindle cell monophasic synovial sarcoma (SS) can be morphologically similar. Angiogenesis is a major factor for tumour growth and metastasis. Our aim was to compare the angiogenic expression profiles of high-risk GIST and spindle cell monophasic SS by histological, immunohistochemical and molecular characterisation of the neovascularisation established between xenotransplanted tumours and the host during the initial phases of growth in nude mice.

**Methods:**

The angiogenic profile of two xenotransplanted human soft-tissue tumours were evaluated in 15 passages in nude mice using tissue microarrays (TMA). Tumour pieces were also implanted subcutaneously on the backs of 14 athymic Balb-c nude mice. The animals were sacrificed at 24, 48, and 96 h; and 7, 14, 21, and 28 days after implantation to perform histological, immunohistochemical, and molecular studies (neovascularisation experiments).

**Results:**

Morphological similarities were apparent in the early stages of neoplastic growth of these two soft-tissue tumours throughout the passages in nude mice and in the two neovascularisation experiments. Immunohistochemistry demonstrated overexpression of pro-angiogenic factors between 24 h and 96 h after xenotransplantation in both tumours. Additionally, neoplastic cells coexpressed chemokines (CXCL9, CXCL10, GRO, and CXCL12) and their receptors in both tumours. Molecular studies showed two expression profiles, revealing an early and a late phase in the angiogenic process.

**Conclusion:**

This model could provide information on the early stages of the angiogenic process in monophasic spindle cell SS and high-risk GIST and offers an excellent way to study possible tumour response to antiangiogenic drugs.

## Introduction

High-grade sarcomas can be implanted into immunodeficient mice, where they grow as xenografts supported by the murine stroma blood supply [1]. In general, transplantations of low-grade tumours fail to establish, with successful xenografts deriving mainly from aggressive neoplasms. Although some properties of the original tumours may not be fully represented, xenografts have become very useful models for preclinical experiments in cancer research [2–5].

In recent years, much research has focused on the role of angiogenesis in tumour development, growth, invasion, and metastasis [6, 7]. It has become clear that tumour angiogenesis is the result of an imbalance between proangiogenic and antiangiogenic factors, the threshold of change in favour of proangiogenesis is considered to be the angiogenic switch. Several angiogenic factors and chemokines related with angiogenic mechanisms have been studied in different tumour types [6, 8, 9]. We recently communicated these findings in a nude mice osteosarcoma model [10] as well as in two high-grade chondrosarcomas (in press) [11]. The angiogenic process presents two different phases of tumour growth. An initial induction phase, in which new unstable vessels are built, followed by a remodelling phase, in which blood vessels are stabilised [12]. At this point, hypoxia occurs and the angiogenic process is activated through the well-known hypoxia-inducible transcription factors (HIF) that induce the expression of several tumour-derived cytokines, such as vascular endothelial growth factors (VEGF) or fibroblast growth factors (FGF) [6] and some chemokines (GRO, CXCL9, and CXCL10) with their respective receptors (CXCR2 and CXCR3) [8, 13, 14]. More recently, the CXCL12/CXCR4 axis was reported to be involved in mediating tumour cell invasion and proliferation and to play an important role in tumour angiogenesis, progression, and metastasis [15, 16]. Moreover, CXCR4 expression has been associated with poor survival in bone and soft-tissue sarcomas [17, 18] and many types of carcinomas [19]. Consequently, considerable interest has been generated in the therapeutic potential of targeting the growth of new vessels (antiangiogenesis) and the capacity to control those that have already been formed (vascular targeting) [13, 20].

Soft-tissue sarcomas are an infrequent group of mesenchymal tumours, they may be high grade and display poor survival [21]. Gastrointestinal stromal tumour (GIST) is the most common primary mesenchymal tumour of the gastrointestinal tract and spans a clinical spectrum from benign to malignant; most cases contain *KIT*- or *PDGFRA*-activating mutations [22]. Mutations in different genes may also be present, [23] although the main prognostic factors are tumour size, mitotic activity, location and capsular invasion [24, 25]. Targeted therapy with imatinib is indicated for high-risk cases and in advanced disease [23].

Synovial sarcoma (SS) is a mesenchymal tumour with a variable degree of epithelial differentiation and a specific chromosomal translocation t(X;18)(p11;q11) that leads to the formation of an *SS18–SSX* fusion gene. The differential diagnosis with a high-grade GIST may be difficult [26, 27]. Both tumour types cause metastasis and display an aggressive behaviour, suggesting that molecular reorganisations such as of *SYT–SSX* gene translocation and *KIT* mutations might be similarly essential for the growth of angiogenic factors. A recent publication described an intra-abdominal monophasic spindle cell SS that mimicked the morphology and immunohistochemistry of a high-risk spindle cell GIST [27].

Several animal models have been used in the study of tumour angiogenesis [12, 14, 28]. Studying angiogenesis through a xenograft model in high-grade sarcomas such as high-risk GIST and synovial sarcomas (SS) may provide a better understanding of this process and increase information regarding potential candidates for effective targeted therapy.

We developed a xenograft nude mice model to clarify the presence of angiogenic factors within the neoformed peritumoral stroma and in the internal tumour blood supply, during the early stages of tumour growth after the transfer into the subcutaneous tissue of the host. To this end, we used two previously established xenotransplanted tumour cell lines of human sarcomas: a high-risk spindle cell GIST and a monophasic spindle cell SS [22, 29].

Our aim was to characterise the markers associated with vasculogenesis using histology, immunohistochemistry, and molecular techniques and to search for similarities that may exist between the two tumours.

## Materials and methods

### Samples

Samples were collected from patients treated at the Hospital Clínic Universitari de Valencia. The GIST came from a 63-year-old male with a gastric mass of approximately 26 × 20 × 35 cm diagnosed as a high-risk spindle cell tumour (Figure 2A). Firstly, the GIST was treated with imatinib (400 mg/day) for six months. The tumour responded partially to targeted therapy and finally resection of the mass was decided upon seven months after diagnosis. No metastasis was seen at the moment of diagnosis, but the patient died of various surgical complications after resection.

The SS came from a 32-year-old male who attended our hospital with a relapse in the right thigh and multiple lung metastases after chemo-radiotherapy. The tumour was approximately 10 × 8 × 8 cm and was diagnosed as monophasic spindle-cell SS, the patient died of tumour progression several months after diagnosis.

Molecular biology studies revealed genomic alterations in both tumours. The GIST had the *KIT* gene mutation and the SS had the typical translocation t(X,18)(*SYT-SSX*).

The tumours were collected for histopathological, ultrastructural, and genetic characterisation at our Pathology Department. The original tumours were transferred subcutaneously on the backs of nude mice (Nu407 and Nu335) and maintained for several generations (passages). In both tumours, we divided the passages into three time periods, early passages (from 1st to 5th passage), middle passages (from 6th to 10th passage) and late passages (from 11th to 15th passage). We calculated the average speed of tumour growth in both nude mice passages (Nu335 and Nu407) according to the formula (15 mm/days to next passage), 15 mm being the approximate tumour size when mice were sacrificed.

Tumour pieces 3–4 mm in size from the early passages of Nu407 and Nu335 were also xenografted subcutaneously on the backs of two sets of athymic Balb-c nude mice (*n* = 14 each). The animals were sacrificed at 24, 48, and 96 h; and 7, 14, 21, and 28 days after implantation (neovascularisation experiments). Tissue samples were fixed in 10% formaldehyde, paraffin-embedded, and haematoxylin and eosin (H&E) staining was performed for histological analysis. Moreover non-fixed samples were collected for molecular analysis. Approval for animal experimentation was obtained from the Ethics Committee of the Universitat de València Estudi General (UVEG).

### Assembly of TMAs

Tissue microarrays were constructed using a Manual Tissue Arrayer (Beecher Instruments, Sun Praire, WI). Two cores (1 mm thick) of each sample were included, with additional cores in cases with diverse morphologic areas. The TMA contained normal tissue controls, original tumour, and the corresponding xenograft passages. The cores were grouped into early transfers 1–5, middle passages 6–10, and late passages 11–15. After assembly, an initial section from each TMA was stained with haematoxylin–eosin to evaluate the viability of the samples. Several 5-mm sections were also prepared for immunohistochemical (IHC) staining. Table 1 summarises the antibodies used for the IHC.

### Immunohistochemistry

IHC was carried out by an indirect peroxidase method on paraffin sections following the same methodology, as we discussed in our previous papers [10] and [11].

### Molecular biology

RNA was extracted from 50 to 200 mg of tumour samples obtained from the NU335 and Nu407 series.

The whole methodology and studied genes (Table 1S) are also discussed in our previous papers [10] and [11].

## Results

### Histological, immunohistochemical and ultrastructural characterisation

**Figure 1.**
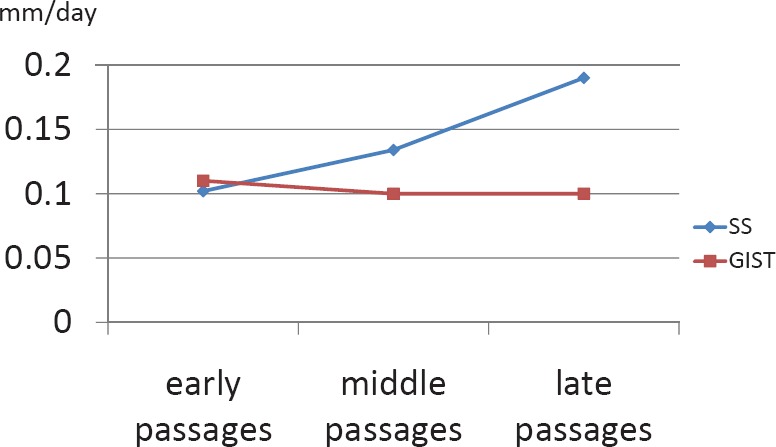
Graphics of speed of growth in both tumours throughout the passages in nude mice.

Comparing speed of growth in murine passages, we observed a similar average growth speed in SS (0.142 mm/day) and in GIST (0.103 mm/day)(Figure 1).

### TMAs

**Figure 2.**
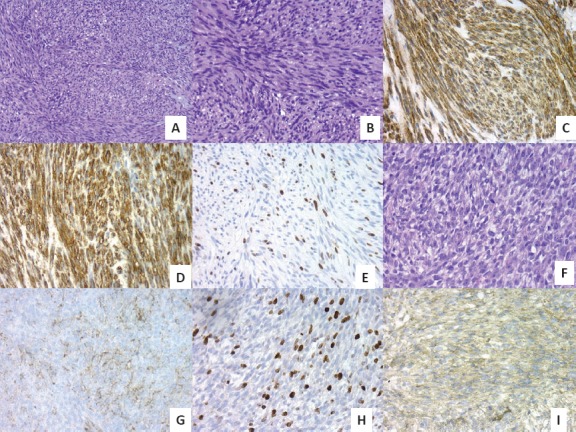
(A) GIST with spindle-cell pattern (H&E, 20X). (B) High-risk GIST with some mitotic figures (H&E, 40X). (C) Intense positivity of DOG-1 in GIST, 40X. (D) Intense positivity of CD34 in GIST, 40X. (E) High proliferative Ki67 index in late passages of GIST, 40X. (F) Monomorphic spindle-cell pattern in SS (H&E, 40X). (G) Mild EMA positivity in SS, 40X. (H) High proliferative Ki67 index in late passages of SS, 40X. (I) Intense VEGF expression in SS 24 h after tumour implantation, 40X.

No morphological changes were observed between passages in GIST; however, a high number of mitoses were clearly observed in the passages in both tumours (Figures 2B and 2F). The IHC study of GIST showed intense expression of vimentin, CD117, DOG1, desmin, and CD34 (Figures 2C and 2D) and was negative for PDGFRα and S-100. Ki67 was expressed in 15% of tumour cells in all cores (Figure 2E).

The IHC study of SS showed positivity for EMA (Figure 2G), cytokeratin (AE1/AE3) and bcl-2. Intense positivity was also revealed for vimentin and weak expression for TLE1 in all passages. Ki67 increased slightly over the passages, being positive in 20% of tumour cells in the last passages (Figure 2H), whereas with GIST it was more constant.

**Figure 3.**
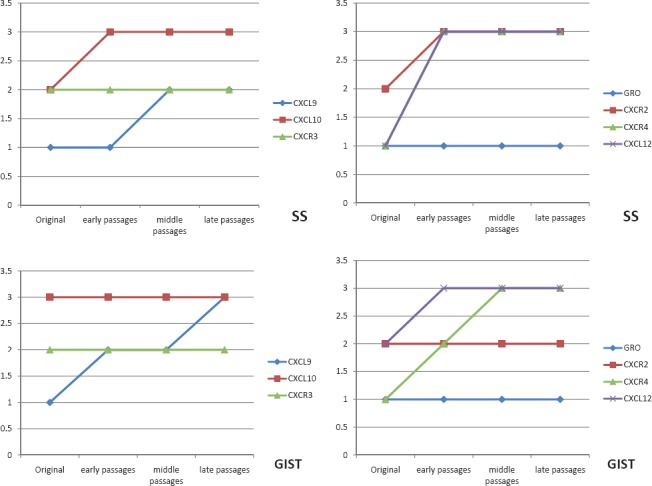
Graphics of chemokine ligand and receptor expression in both tumours throughout the passages in nude mice.

**Figure 4.**
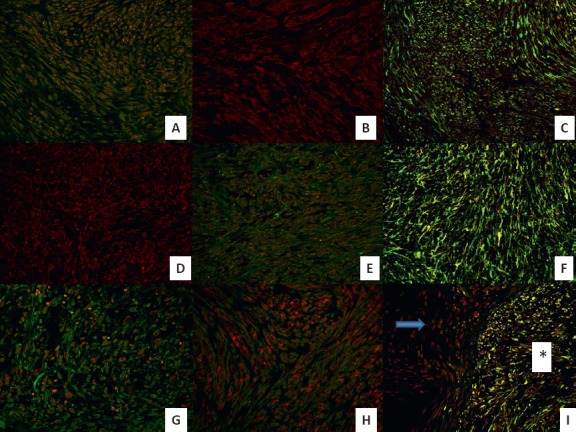
Immunofluorescent expression of chemokines and their receptors in GIST and SS throughout the early, middle, and late passages and in the neovascularisation experiment. (A) Double-immunofluorescence staining shows coexpression of chemokine ligand CXCL10 (red) and its receptor CXCR3 (green) in GIST tumour cells in early passages (40X). (B) Immunofluorescence staining shows expression of chemokine ligand CXCL9 (red) in GIST tumour cells in later passages (40X). (C) double-immunofluorescence staining shows coexpression of chemokine ligand CXCL12 (green) and its receptor CXCR4 (red) in GIST tumour cells in middle passages (40X). (D) Immunofluorescence staining shows expression of chemokine ligand GRO (red) in SS tumour cells in middle passages (40X). (E) double-immunofluorescence staining shows coexpression of chemokine ligand CXCL10 (red) and its receptor CXCR3 (green) in SS tumour cells in late passages (40X). (F) Double-immunofluorescence staining shows high coexpression of chemokine ligand CXCL12 (green) and its receptor CXCR4 (red) in SS tumour cells in late passages (40X). (G) Double-immunofluorescence staining shows coexpression of chemokine ligand GRO (red) and its receptor CXCR2 (green) in SS tumour cells 24 h after xenografting (40X). (H) Double-immunofluorescence staining shows coexpression of chemokine ligand GRO (red) and its receptor CXCR2 (green) in GIST tumour cells in the control tumour (40X). (I) Double-immunofluorescence staining shows coexpression of chemokine ligand CXCL12 (green) and its receptor CXCR4 (red) in GIST tumour cells two weeks after xenotransplantation (40X), observe the different expression between murine stroma (arrow) and tumour cells (asterisk).

Double-immunofluorescence staining demonstrated that chemokine ligand expression in general was slightly higher in the xenograft passages than in the original tumour (Figure 3). There were very few differences between the two sarcomas with regard to chemokine expression profile. CXCL10 was constantly high in both tumours and GRO was mildly expressed in all passages (Figures 4A, 4D, and 4E). CXCL9 increased in both tumours over the passages (Figure 4B). Their receptors CXCR2 and CXCR3 were constantly expressed in all passages, with CXCR2 presenting a higher expression in SS. Finally, the CXCL12/CXCR4 axis was constantly overexpressed in all passages in both tumours (Figures 4C and 4F).

### Neovascularisation experiments

In our neovascularisation experiments, during the first hours after xenografting, peritumoral haemorrhagic areas with inflammatory infiltration compounded by lymphocytes, plasma cells, neutrophils, karyorrhectic, and apoptotic figures were observed in SS and GIST. Small capillaries surrounded the xenograft associated with mesenchymal angioblastic and non-angioblastic cells included in a loose matrix.

Patchy hypoxic necrosis in SS appeared within the first 96 h after implantation, reaching a peak extension in the third week. The SS presented characteristic adipose tissue infiltrate and peritumoral skeletal muscle mouse fibres, but without the pseudocapsule observed in GIST. In GIST, the massive necrosis appeared during the first week, earlier than in the SS. After the fourth week, the histological picture of the GIST was re-established, with features similar to those of the human control, re-establishing also the amount of mitoses and the remission of necrosis, which became patchy and scant. During the third week after xenografting, the inflammatory component decreased. At this time, newly formed capillary vessels were remodelled and penetrated or sprouted into the tumour.

Areas of massive necrosis were associated with a lower proliferative index in both tumours. Ki67 was lower in the early stages after tumour xenografting in both tumours. In the last weeks, the increase in Ki67 expression was also inversely correlated with HIF1α in both neoplasms.

Angiogenic factors represented by the VEGF family and their receptors presented a different expression profile in the two tumours. In SS, maximum VEGF positivity presented 24 h after implantation and was also expressed in the extracellular matrix, while VEGF positivity was lower in GIST and appeared 96 h after xenografting (Figure 2I). VEGFR2 presented a similar expression profile to its ligand, and VEGFR3 was the most positive receptor in both tumours. HIF1α expression was slightly higher and more constitutively expressed in SS than GIST

Double-immunofluorescence staining showed chemokine expression (CXCL9, CXCL10 and GRO) in the tumour cell cytoplasm/nucleus and deposited in the extracellular matrix. This was also the case for their receptors (CXCR3 and CXCR2) (Figures 4G and 4H). Chemokine ligand expression was higher during the first 48 h in GIST; however, it appeared later in SS where peak expression occurred during the first week. The CXCL12/CXCR4 axis showed an intense coexpression in both sarcomas at all times throughout the experience. Interestingly, we observed that murine peritumoral stroma expressed CXCR4 but not CXCL12 in the two tumour xenografts (Figure 4I). It is worth mentioning that the chemokine receptors were expressed more constantly at all times in comparison with their ligands.

### qRT-PCR low-density arrays of angiogenesis-related genes

**Figure 5.**
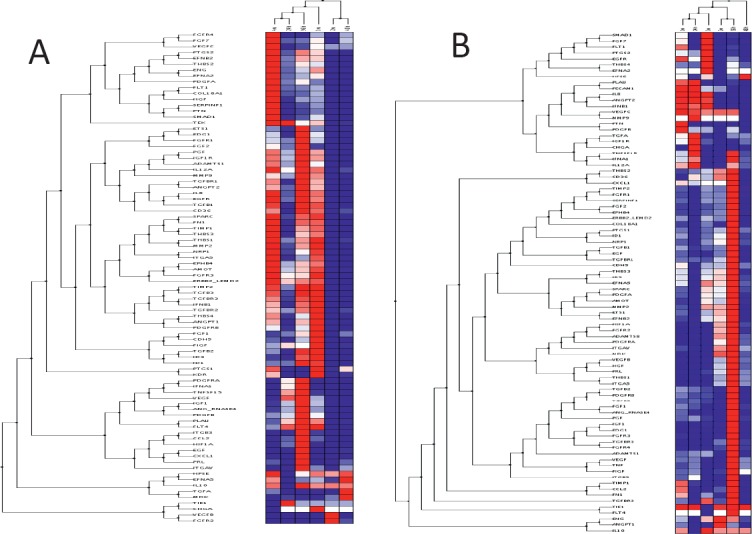
Cluster tree of genes related to angiogenesis by qRT-PCR obtained from the Nu335 (SS) (A) and Nu407 (GIST) (B) series using distance correlation and applying a linear correlation coefficient at different times. Overexpressed genes are shown in dark red, underexpressed genes in dark blue and no change in white. Samples from third week in SS, and fourth week in GIST were not available for analysis (Table 2S and 3S).

Gene expression profiles (Figure 5) in GIST were similar at 24 h and 7 days but differed from those observed at 48 h, 14 and 21 days. However, SS expression profiles were similar at 24 h and 28 days, differing from those at 48 h and 14 days. In GIST, the early phase appeared 96 h after xenografting and was characterised by the overexpression of genes clearly involved in angiogenesis induction, including VEGF, PDGFA, PDGFB, VEGFC, and their receptors. In contrast, the earlier phase in SS occurred during the first week after xenografting. Finally, in GIST, the late phase of the angiogenic process (remodelling phase) appeared during the first week after xenografting, while in SS, this phase appeared later in the fourth week.

RNA samples corresponding to the third week of SS and to the fourth week of GIST were not viable for analysis.

## Discussion

Angiogenesis is critical for the growth and metastasis of tumours. Early in tumorigenesis, an angiogenic switch is activated by hypoxia, promoting the expression of pro-angiogenic growth factors, such as the VEGF family, its receptors, and HIF1α among others [7]. Recent publications have highlighted the difficulties in differentiating between GIST and monophasic intra-abdominal SS, where molecular mutations are sometimes the only distinguishing feature [27]. It has been suggested that it would be particularly informative to explore possible relationships between the presence of vasculogenic structures and the response to antiangiogenesis therapy [30, 31]. Furthermore, it is interesting to speculate that antiangiogenic therapy may result in a selective growth advantage for cells exhibiting vasculogenic mimicry and vascular co-option, promoting drug-induced resistance [30, 31].

HIF1α is a principal regulator of cellular and systemic homeostatic response to hypoxia as it activates many genes, including those involved in angiogenesis [6]. In our model, HIF1α was overexpressed in the early stages, indicating that the angiogenic process is constitutively active in the xenotransplanted tumour. HIF1α plays an important role in angiogenic induction and remodelling phases and in an increased VEGF expression [32]. Some recent studies of GIST and chondrosarcoma have shown a correlation between the expression of angiogenic markers, such as VEGF and microvessel density, and a worse prognosis [33, 34], suggesting that the development of antiangiogenic chemotherapy might be useful. However, in other tumours, such as osteosarcoma, microvessel density seems to be associated with a longer overall and relapse-free survival [34, 35]. Imatinib continues to be used as a first-line medical treatment for advanced GIST, although resistance and non-response sometimes appear. Sunitinib and regorafenib, antiangiogenic drugs, are used as second- and third-line therapies, respectively, and are given in imatinib-resistant GIST cases [23]. Volumetric growth and the development of metastases in cases of GIST appear to be related to the development of a new vascular network [36]. The importance of vascularisation in the context of GIST is the action mechanism of the second-generation drug sunitinib, which is based on the blockade of VEGF activity along with tyrosine kinase receptor blockade that has been used with success in some GIST patients [37, 38].

Anthracyclines and ifosfamide, either alone or in combination, are the gold standard treatments for advanced SS [39]. However, after failure of conventional first-line cytotoxic chemotherapy, available treatment options are severely limited because of a high risk-to-benefit ratio in terms of patient tolerability and survival. Recently, it has been demonstrated that pazopanib is a feasible option for patients who have been heavily pre-treated for metastatic SS [40].

Cluster analysis performed on our qRT-PCR expression studies revealed two additional groups of genes clearly separated into two stages, corresponding to early angiogenic induction where VEGF and PDGF family genes among others play an important role, and the later remodelling phases where other angiogenic genes are overexpressed. Apparently the high-risk GIST behaved biologically as high-grade sarcoma in the passages and neovascularisation experiments similar to our previous molecular results [10]. However, induction and remodelling phases of SS appeared later than GIST and other high-grade bone tumours [10]. This difference may be related to a different sensibility and response to antiangiogenic drugs, with GIST being more sensitive. Nevertheless, we cannot be sure that this difference will have any biological translation.

In addition to angiogenic factors, chemokines also play an important role during angiogenic induction. The coexpression of ligands and chemokine receptors in neoplastic cells and extracellular matrix suggests that autocrine and paracrine stimulation by the tumour cells results in production of angiogenic factors in response to hypoxia during the first stages of tumour growth, as reported in other neoplastic and non-neoplastic conditions [8, 12, 30, 41]. Moreover, we found a correlation between high chemokine ligand expression and hypoxic necrosis in both tumours. Few studies of chemokines in SS and GIST have been made [42, 43]. CXCR4 expression has been related with poor prognosis in patients with bone and soft-tissue sarcomas in a meta-analysis [17]. High expression of CXCL12/CXCR4 was observed in all passages of both tumours and in the neovascularisation experiment, this could be related with their aggressive clinical behaviour. The CXCL12/CXCR4 axis is related to mediating tumour cell invasion and proliferation and plays an important role in tumour angiogenesis, progression and metastasis [44]. CXCL12/CXCR4 is overexpressed by tumour cells, but not by murine stromal peritumoral cells which only produce CXCR4. Perhaps CXCL12 induces murine stromal cells to generate new vessels in a paracrine effect and may be a good objective for targeted therapy to reduce tumour growth.

## Conclusions

This model provides information on the early stages of the angiogenic process in monophasic spindle-cell SS and high-risk GIST. We suggest that different angiogenic molecular profiles could predict different biological and clinical behaviour and determine the response to antiangiogenic treatment. We also demonstrate the importance of chemokine expression as a therapeutic target of tumour growth.

The fact that angiogenesis is a dynamic, changing and multistep process over time should be taken into consideration when developing future therapeutic strategies in soft-tissue tumours.

## Conflict of Interest

The authors declare that they have no conflict of interest.

## Figures and Tables

**Table 1.** Antibodies used in the experiences.

Marker	Clonality (Clon)	Location	Dilution	Manufacturer
Ki-67	Monoclonal (MIB-1)	Nuclear	1/50	Dako
HIF1 α	Monoclonal (HI α 67)	Cytoplasm	1/500	Chemicon
VEGF	Monoclonal (VG 1)	Cytoplasm	1/100	Neomarkers
VEGFR1	Polyclonal (rabbit)	Cytoplasm/membrane	1/400	Santa Cruz
VEGRF2	Polyclonal (rabbit)	Cytoplasm/membrane	1/400	Santa Cruz
VEGFR3	Polyclonal (rabbit)	Cytoplasm/membrane	1/400	Santa Cruz
PDGFR α	Polyclonal (rabbit)	Cytoplasm	1/100	Santa Cruz
FGF 2	Polyclonal (rabbit)	Cytoplasm	1/200	Santa Cruz
VE- CAD	Polyclonal (goat)	Cytoplasm/membrane	1/50	Santa Cruz
CXCL9	Polyclonal (goat)	Cytoplasm	1/100	R&DSystems
CXCL10	Polyclonal (goat)	Nuclear/cytoplasm	1/100	R&DSystems
GRO	Monoclonal (31716)	Cytoplasm	1/200	R&DSystems
CXCL12	Monoclonal(79018)	Cytoplasm	1/60	R&DSystems
CXCR3	Monoclonal (2Ar1)	Cytoplasm/membrane	1/200	Abcam
CXCR2	Polyclonal(mouse)	Cytoplasm/membrane	1/20	BioLegend
CXCR4	Monoclonal(44716)	Nuclear	1/150	R&DSystems
Vimentine	Monoclonal (V9)	Cytoplasm	1/300	Novocastra
DOG-1	Monoclonal (BV10)	Cytoplasm	1/50	DBS
CD117	Polyclonal (rabbit)	Membrane/cytoplasm	1/100	Dako
TLE-1	Polyclonal (rabbit)	Nuclear	1/50	Santa Cruz
CD34	Monoclonal (QBEnd10)	Cytoplasm	pre-diluted	Dako
S-100	Polyclonal (rabbit)	Cytoplasm	1/2000	Dako
Desmin	Monoclonal (D33)	Cytoplasm	pre-diluted	Dako
EMA	Monoclonal (E29)	Cytoplasm/membrane	1/200	Dako
CK(AE1/AE3)	Monoclonal (AE1/AE3)	Cytoplasm/membrane	1/100	Dako
BCL-2	Monoclonal (3.1)	Nuclear	1/50	Novocastra

**Table 1.** Supplementary. Genes and assays included in the low-density array for the study of the expression of angiogenic factors by quantitative RT-PCR.

S.No	Gene symbol	Assay references (Applied Biosystems)	Amplicon size (pb)	OMIM
1	*AMOT (angiomotin)*	Hs00611096_m1	65	300410
2	*ANG (angiogenina)*	Hs00265741_s1	91	105850
3	*ANGPT1 (angiopoietin-1)*	Hs00181613_m1	87	601667
4	*ANGPT2 (angiopoietin-2)*	Hs00169867_m1	73	601922
5	*CHGA (Vasostatin/Chromogranin-A)*	Hs00154441_m1	115	118910
**Growth factors and receptors**				
6	*Ephrin 2A (EFNA2)*	Hs00154858_m1	89	602756
7	*Ephrin-A5 (EFNA5)*	Hs00157342_m1	98	601535
8	*Ephrin-B2 (EFNB2)*	Hs00187950_m1	63	600527
9	*EPHB4 (Ephrin B4)*	Hs00174752_m1	82	600011
10	*FGF1 (aFGF)*	Hs00265254_m1	65	131220
11	*FGF2 (bFGF)*	Hs00266645_m1	82	134920
12	*FGF4*	Hs00173564_m1	53	164980
13	*FGF6*	Hs00173934_m1	64	134921
14	*FGF7*	Hs00384281_m1	85	148180
15	*FGFR1*	Hs00241111_m1	81	136350
16	*FGFR2 (KGFR)*	Hs00240792_m1	77	176943
17	*FGFR3*	Hs00179829_m1	104	134934
18	*FGFR4*	Hs00242558_m1	74	134935
19	*PDGFA*	Hs00234994_m1	93	173430
20	*PDGFB*	Hs00234042_m1	80	190040
21	*PDGFRA*	Hs00183486_m1	92	173490
22	*PDGFRB*	Hs00182163_m1	86	173410
23	*PF4 (Platelet factor 4)*	Hs00236998_m1	86	173460
24	*TGFA*	Hs00177401_m1	95	190170
25	*TGFB1*	Hs00171257_m1	63	190180
26	*TGFB2*	Hs00234244_m1	92	190220
27	*TGFB3*	Hs00234245_m1	75	190230
28	*TGFBR1*	Hs00610319_m1	126	190181
29	*TGFBR2*	Hs00234253_m1	70	190182
30	*TGFBR3*	Hs00234257_m1	60	600742
31	*FIGF (VEGFD)*	Hs00189521_m1	130	300091
32	*FLK1 (KDR)*	Hs00176676_m1	84	191306
33	*FLT1 (VEGFR2)*	Hs00176573_m1	55	165070
34	*PGF*	Hs00182176_m1	56	601121
35	*VEGF*	Hs00173626_m1	77	192240
36	*VEGFB*	Hs00173634_m1	69	601398
37	*VEGFC*	Hs00153458_m1	126	601528
38	*FLT4 (VEGFR3)*	Hs01047679_m1	89	136352
39	*CD36*	Hs00169627_m1	77	173510
40	*EDG1*	Hs00173499_m1	102	601974
41	*EGF*	Hs00153181_m1	95	131530
42	*EGFR*	Hs00193306_m1	69	131550
43	*GRO1 (CXCL1)*	Hs00236937_m1	70	155730
44	*HGF (Scatter factor)*	Hs00300159_m1	92	142409
45	*IGF1*	Hs00153126_m1	70	147440
46	*IGF1R*	Hs00181385_m1	77	147370
47	*TEK (Tie-2)*	Hs00176096_m1	82	600221
48	*TIE1*	Hs00178500_m1	74	600222
**Cytokines and chemokines**				
49	*CSF3*	Hs00236884_m1	78	138970
50	*IFNA1*	Hs00256882_s1	115	147660
51	*IFNB1*	Hs00277188_s1	134	147640
52	*IFNG*	Hs00174143_m1	79	147570
53	*IL10*	Hs00174086_m1	119	124092
54	*IL12A*	Hs00168405_m1	67	161560
55	*IL8*	Hs00174103_m1	101	146930
56	*MDK*	Hs00171064_m1	102	162096
57	*NRP1 (neuropilin-1)*	Hs00826129_m1	97	602069
58	*PRL (prolactin)*	Hs00168730_m1	76	176760
59	*PTN (pleiotrophin)*	Hs00383235_m1	76	162095
60	*SCYA2*	Hs00234140_m1	101	158105
61	*SPARC*	Hs00277762_m1	122	182120
62	*TNFA*	Hs00174128_m1	80	191160
63	*TNFSF15 (VEGI)*	Hs00353710_s1	97	604052
**Adhesion molecules**				
64	*CDH5 (VE-Cadherin)*	Hs00174344_m1	66	601120
65	*ITGA5 (integrin a5)*	Hs00233743_m1	86	135620
66	*ITGAV (integrin aV)*	Hs00233808_m1	64	193210
67	*ITGB3*	Hs00173978_m1	89	173470
68	*PECAM1 (CD31)*	Hs00169777_m1	65	173445
**Matrix Proteins, Proteases and Inhibitors**				
69	*ADAMTS1 (METH1)*	Hs00199608_m1	68	605174
70	*ADAMTS8 (METH2)*	Hs00199836_m1	59	605175
71	*COL18A1 (LOC51695/endostatin)*	Hs00181017_m1	72	120328
72	*FN1 (fibronectin)*	Hs00415006_m1	86	135600
73	*HPSE (heparanase)*	Hs00180737_m1	59	604724
74	*MMP2*	Hs00234422_m1	83	120360
75	*MMP9*	Hs00234579_m1	54	120361
76	*MSR1*	Hs00234012_m1	80	153622
77	*PLAU (uPA)*	Hs00170182_m1	104	191840
78	*SERPINB5 (maspin)*	Hs00184728_m1	104	154790
79	*SERPINF1 (PEDF)*	Hs00171467_m1	88	172860
80	*THBS1*	Hs00170236_m1	109	188060
81	*THBS2*	Hs00170248_m1	87	188061
82	*THBS3*	Hs00200157_m1	85	188062
83	*THBS4*	Hs00170261_m1	98	600715
83	*TIMP1*	Hs00171558_m1	104	305370
85	*TIMP2*	Hs00234278_m1	73	188825
**Transcription factors**				
86	*ERBB2*	Hs00397754_m1	58	164870
87	*ETS1*	Hs00428287_m1	92	164720
88	*HIF1A*	Hs00153153_m1	76	603348
89	*ID1*	Hs00357821_g1	62	600349
90	*ID3*	Hs00171409_m1	129	600277
91	*MADH1 (SMAD1)*	Hs00195432_m1	67	601595
**Other related genes**				
92	*ENG (endoglin)*	Hs00164438_m1	81	131195
93	*PTGS1 (cox-1)*	Hs00168776_m1	109	176805
94	*PTGS2 (cox-2)*	Hs00153133_m1	75	600262
95	*B2M (Housekeeping)*	Hs99999907_m1	75	109700
96	*18S (Housekeeping)*	Hs99999901_s1	187	180473

**Table 2.** Supplementary. 2-^DDCt^ values corresponding to the Nu335 series.

Gene_Symbol	24 h	48 h	96 h	1 week	2 week	3 week	4 week
**ADAMTS1-Hs00199608_m1**	0.7959605	0.03487536	2.2625384	1.1141776	0.010976258		1.7567259
**AMOT-Hs00611096_m1**	0.26376235	0.05707947	0.37297338	0.5533971	5.48E-04		0.6260453
**ANG;RNASE4-Hs00265741_s1**	8.801705	0.049484883	23.473007	7.5595713	0.015574286		2.9783483
**ANGPT1-Hs00181613_m1**	0.009880596	0.05084049	1.1490225	1.854033	0.016000934		1.1941226
**ANGPT2-Hs00169867_m1**	3.6326275	0.03473391	16.737255	10.326439	0.01093174		10.955148
**CCL2-Hs00234140_m1**	2.683348	0.16359264	13.288964	2.8040843	0.05148721		1.0333282
**CD36-Hs00169627_m1**	0.053531617	0.2754463	1.3808194	1.1198069	0.0866907		1.021473
**CDH5-Hs00174344_m1**	0.31543005	0.1952118	1.5071367	3.5636954	0.06143865		0.7977747
**CHGA-Hs00154441_m1**	NV	NV	NV	32.096264	NV		4.5581665
**COL18A1-Hs00181017_m1**	0.23082852	5.42E-04	0.277198	0.50039613	1.71E-04		1.3184813
**CXCL1-Hs00236937_m1**	27.300922	NV	89.66844	32.197247	NV		NV
**EDG1-Hs00173499_m1**	0.059957597	0.006040307	1.2171141	0.56891876	0.001901055		0.5275882
**EFNA2-Hs00154858_m1**	1.2024206	0.030415064	2.0298295	2.827156	0.009572477		5.3208594
**EFNA5-Hs00157342_m1**	0.24109949	2.847728	0.9345426	1.2443724	0.58063984		2.1034224
**EFNB2-Hs00187950_m1**	0.23342909	0.001960718	0.57025087	0.5076365	6.17E-04		0.87659615
**EGF-Hs00153181_m1**	0.038981758	0.20058018	1.7117009	0.016707698	0.063128226		0.013953778
**EGFR-Hs00193306_m1**	1.1801668	0.008366582	5.0673194	3.822592	0.002633199		5.079238
**ENG-Hs00164438_m1**	0.20527008	0.016204517	0.41111425	0.6621962	0.005100017		0.94549626
**EPHB4-Hs00174752_m1**	0.21828675	0.004649402	0.34418315	0.44173142	0.004203849		0.56949294
**ERBB2;LEMD2-Hs00397754_m1**	0.46552873	0.00398609	0.5910703	0.673706	0.001254535		0.79151744
**ETS1-Hs00428287_m1**	0.49607438	0.15065551	4.2109904	1.3082498	0.08166007		1.3172712
**FGF1-Hs00265254_m1**	NV	41.08322	72.76203	120.863304	11.979111		26.906906
**FGF2-Hs00266645_m1**	0.17037809	0.04496078	0.62800163	0.09589044	0.014150422		0.33385146
**FGF7-Hs00384281_m1**	42.009033	89.45992	52.361824	112.07813	0.17391264		214.21053
**FGFR1-Hs00241111_m1**	0.68052554	5.25E-04	2.3998103	1.0269998	1.65E-04		1.056858
**FGFR2-Hs00240792_m1**	6.43E-04	0.003308094	5.33E-04	2.76E-04	0.019054554		2.30E-04
**FGFR3-Hs00179829_m1**	0.47414583	0.010906449	0.5086395	0.9106528	0.003432566		0.87494045
**FGFR4-Hs00242558_m1**	0.003453373	0.017769288	0.002865036	0.01827872	0.005592495		0.043755062
**FIGF-Hs00189521_m1**	1.0680918	0.28433153	0.96531004	1.8435278	0.08948714		0.71032244
**FLT1-Hs00176573_m1**	14.741555	0.31529078	18.3541	25.0587	0.099230886		66.39617
**FLT4-Hs01047679_m1**	1.6021613	0.22230674	1.8908751	1.8908751	0.06996619		0.638229
**FN1-Hs00415006_m1**	0.009271171	0.001936899	0.31474605	0.31758147	3.59E-05		0.38421825
**HGF-Hs00300159_m1**	0.74494153	0.28053638	2.331105	2.4146729	0.088292696		6.643718
**HIF1A-Hs00153153_m1**	5.79E-04	2.21E-04	0.002376824	1.50E-04	6.96E-05		1.54E-05
**HPSE-Hs00180737_m1**	0.49950182	2.8662815	1.926133	2.6158357	2.3470185		3.2462463
**ID1-Hs00357821_g1**	0.73769003	9.48E-04	2.9761086	2.6793191	2.98E-04		0.79354596
**ID3-Hs00171409_m1**	0.31624177	0.004542778	3.55751	4.1943107	0.00142974		0.5460467
**IFNA1-Hs00256882_s1**	17.625673	0.022310615	33.60883	2.8208911	0.0561009		0.001552084
**IFNB1-Hs00277188_s1**	270.98575	10.319205	2313.754	2928.3418	0.08540048		1875.1154
**IGF1-Hs00153126_m1**	0.8666961	0.050800312	2.1075785	0.8580654	0.015988288		0.26206714
**IGF1R-Hs00181385_m1**	0.38215917	0.010849698	0.93247163	0.640436	0.003414705		0.7276512
**IL10-Hs00174086_m1**	458.27228	NV	3026.768	2293.5767	NV		2484.658
**IL12A-Hs00168405_m1**	0.567523	0.22941716	0.98875076	0.84497744	0.07220404		1.0499607
**IL8-Hs00174103_m1**	16.041344	0.13745503	85.10393	68.07324	0.043260965		86.558304
**ITGA5-Hs00233743_m1**	0.04292659	0.004046804	0.22571458	0.5543176	0.001273643		0.6485964
**ITGAV-Hs00233808_m1**	3.62E-04	0.001865129	0.005105441	0.001578465	5.87E-04		1.30E-04
**ITGB3-Hs00173978_m1**	0.63354474	0.15661171	5.783896	0.8021871	0.049290113		0.25538987
**KDR-Hs00176676_m1**	0.049058992	0.25243247	0.04070101	1.8342797	0.079447605		1.1495711
**MDK-Hs00171064_m1**	5.30E-05	2.73E-04	4.40E-05	2.27E-05	8.59E-05		1.90E-05
**MMP2-Hs00234422_m1**	0.19907643	1.38E-04	0.87531877	1.2991811	4.35E-05		1.2406509
**MMP9-Hs00234579_m1**	2.7669928	0.17505844	7.6940885	5.5476108	0.055095818		4.2932944
**NRP1-Hs00826129_m1**	0.12803793	7.99E-04	0.4838971	0.84648466	0.003289067		0.93841285
**PDGFA-Hs00234994_m1**	0.4389812	0.002506821	0.8385384	0.43000203	7.89E-04		1.611485
**PDGFB-Hs00234042_m1**	1.2534354	0.016952496	2.7536328	1.3462225	0.005335427		0.8673197
**PDGFRA-Hs00183486_m1**	0.019641923	2.46E-04	0.037229206	0.003201435	7.74E-05		5.76E-04
**PDGFRB-Hs00182163_m1**	0.26188913	0.035274055	1.1167214	2.384623	0.011101739		1.1476152
**PGF-Hs00182176_m1**	2.9858983	0.024673844	7.7015076	5.086536	0.007765553		6.028427
**PLAU-Hs00170182_m1**	1.1980854	0.27836916	1.982732	1.4934684	0.08761061		0.44911763
**PRL-Hs00168730_m1**	0.106087364	0.54587126	2.3486202	0.04546936	0.17180106		0.4713016
**PTGS1-Hs00168776_m1**	0.053466685	0.2751122	0.04435778	0.4627111	0.08658555		0.39559445
**PTGS2-Hs00153133_m1**	345.7538	0.5678802	1413.4844	1064.7853	0.1787279		1878.3756
**PTN-Hs00383235_m1**	0.18134442	0.007857767	0.64166147	1.232515	0.002473061		4.575156
**SERPINF1-Hs00171467_m1**	0.073242776	2.50E-04	0.38210613	0.7587081	7.86E-05		2.3391302
**SMAD1-Hs00195432_m1**	1.1732796	0.002044792	3.2746916	8.598647	6.44E-04		18.83404
**SPARC-Hs00277762_m1**	0.1108581	0.001018303	0.63713557	0.6971213	2.43E-04		0.76808304
**TEK-Hs00176096_m1**	0.68615204	0.10372496	0.37136102	0.4196441	0.032645162		0.68975556
**TGFA-Hs00177401_m1**	0.007058851	4.596679	0.19149838	0.26590338	0.105379626		1.2052153
**TGFB1-Hs00171257_m1**	0.10877489	0.004435655	1.9193434	1.6530409	0.001396026		1.7242657
**TGFB2-Hs00234244_m1**	0.13737491	0.001103383	3.099895	3.3531804	3.47E-04		1.0426474
**TGFB3-Hs00234245_m1**	0.15789403	0.00436108	1.0450748	1.0556368	0.001372555		0.9004825
**TGFBR1-Hs00610319_m1**	0.09502458	0.004009001	2.2182114	1.4024074	0.001261745		1.632517
**TGFBR2-Hs00234253_m1**	31.741417	14.806774	75.4627	98.78798	0.070976205		58.946075
**TGFBR3-Hs00234257_m1**	0.0168747	0.002223517	0.10899068	0.1265091	7.00E-04		0.093545
**THBS1-Hs00170236_m1**	0.02627843	0.013971536	0.05666908	0.07892739	0.004397236		0.080069
**THBS2-Hs00170248_m1**	0.24877942	0.00247538	0.5332035	0.46211198	7.79E-04		0.99361634
**THBS3-Hs00200157_m1**	0.057871778	0.010964321	0.81936586	1.063532	0.00345078		1.3852974
**THBS4-Hs00170261_m1**	0.25605658	0.041287363	1.055909	2.2569547	0.012994295		1.6387945
**TIE1-Hs00178500_m1**	14.531259	NV	NV	8.991988	NV		5.8403964
**TIMP1-Hs00171558_m1**	0.09594396	0.001097487	1.7285247	2.1242523	3.45E-04		2.5862403
**TIMP2-Hs00234278_m1**	0.23860645	0.001554409	0.8069738	0.8414052	4.48E-05		0.7541748
**TNFSF15-Hs00353710_s1**	8.819573	0.09758525	13.036365	1.5936899	0.030712826		0.28507906
**VEGF-Hs00173626_m1**	10.268918	0.004262844	12.111028	4.5594625	0.001341637		0.90244776
**VEGFB-Hs00173634_m1**	0.04733373	0.001347965	0.0785328	0.041420598	1.1771686		0.013903745
**VEGFC-Hs00153458_m1**	85.88848	NV	130.90923	236.45436	NV		379.6854

**Table 3.** Supplementary. 2-^DDCt^ values corresponding to the Nu407 series.

Gene_Symbol	24 h	48 h	96 h	1 week	2 week	3 week	4 week
**ADAMTS1-Hs00199608_m1**	0.96953326	0.43141022	2.2340128	0.7876811	0.40883076	1.062367	
**ADAMTS8-Hs00199836_m1**	0.032204915	0.2006014	0.98537743	0.010386795	0.6983311	0.012592207	
**AMOT-Hs00611096_m1**	0.21026719	0.31007886	1.262663	0.33543622	0.6504736	0.77168876	
**ANG;RNASE4-Hs00265741_s1**	2.2507687	1.7311066	18.238356	4.7330155	1.158861	1.4419116	
**ANGPT1-Hs00181613_m1**	0.16360757	0.15879923	0.43314987	0.4038482	1.0788392	0.241588	
**ANGPT2-Hs00169867_m1**	609.7327	0.12752116	4.6148005	571.9398	4.092888	540.53394	
**CCL2-Hs00234140_m1**	0.8783502	0.7705368	2.7677026	2.3633595	1.1406118	0.93977857	
**CD36-Hs00169627_m1**	0.26223594	0.104032315	0.3904494	0.11094416	0.32380894	0.2182351	
**CDH5-Hs00174344_m1**	0.010893709	0.17642228	0.64253837	0.34452096	0.26403275	0.24607353	
**CHGA-Hs00154441_m1**	647.80444			502.62183		500.9494	
**COL18A1-Hs00181017_m1**	0.93923825	0.9046015	2.5215695	1.1123142	1.0048001	1.3427103	
**CXCL1-Hs00236937_m1**	112.537384	16.713638	234.58054	139.9429			
**EDG1-Hs00173499_m1**			70.72058	11.466081		13.262654	
**EFNA2-Hs00154858_m1**					16.562565	18.947788	
**EFNA5-Hs00157342_m1**	0.15907577	0.3788561	1.2844433	0.6810755	0.79165244	0.9987567	
**EFNB2-Hs00187950_m1**	0.16524926	0.11053204	0.9572704	0.2282536	0.58183163	0.4981927	
**EGF-Hs00153181_m1**	0.33341965	0.3479269	2.3004372	0.67772156	0.81617755	0.26688036	
**EGFR-Hs00193306_m1**	3.6581511	0.79179686	2.7238734	6.1475854	0.58937544	6.7861004	
**ENG-Hs00164438_m1**	0.13470362	0.27517623	0.8537688	0.5037209	1.0865717	0.72379035	
**EPHB4-Hs00174752_m1**	0.36123836	0.46923497	2.6141968	0.6678707	1.0167493	0.82676744	
**ERBB2;LEMD2-Hs00397754_m1**	0.45529452	0.43083453	1.3008044	0.7128512	0.68019813	0.7796248	
**ETS1-Hs00428287_m1**	0.21070047	0.21789578	1.0790489	0.3943694	0.7580288	0.54431254	
**FGF1-Hs00265254_m1**	3.4499087	2.8318844	12.711818	4.67827	1.9497786	1.498922	
**FGF2-Hs00266645_m1**	0.3093668	0.2914567	1.3103998	0.42455202	0.5530541	0.4609033	
**FGF7-Hs00384281_m1**	48.65966	19.154825	38.67718	518.2895	8.4551935	979.51294	
**FGFR1-Hs00241111_m1**	0.3395128	0.3969545	1.2806027	0.48343667	0.6041856	0.6130072	
**FGFR2-Hs00240792_m1**	0.010584445	0.09899647	0.89178413	0.004572303	0.5568605	0.006664489	
**FGFR3-Hs00179829_m1**	0.5357339	0.48000842	8.065524	1.1046512	0.70578426	0.27442563	
**FGFR4-Hs00242558_m1**	0.009197846	0.45745876	3.604345	0.09207129	0.52055645	0.058838986	
**FIGF-Hs00189521_m1**	0.6024093	1.0161419	1.9958794	0.40206885	0.2503804	0.003528847	
**FLT1-Hs00176573_m1**	4.551344	1.6045651	10.260301	53.115604	2.5825722	65.7581	
**FLT4-Hs01047679_m1**			35.10392			17.77765	
**FN1-Hs00415006_m1**	0.8966703	1.0181764	4.5080233	3.212114	1.1104013	2.1068652	
**HGF-Hs00300159_m1**	0.014922882	0.55347687	2.7260602	0.019646	1.1644697	0.05273539	
**HIF1A-Hs00153153_m1**	0.12633125	0.2151466	1.1821109	0.1177659	0.7790458	0.045135695	
**HPSE-Hs00180737_m1**	2.0230112	5.3897333	2.5039659	3.5169048	1.5538273	3.7573454	
**ID1-Hs00357821_g1**	1.0472269	1.8223221	6.7548165	2.972951	3.8188794	2.4504051	
**ID3-Hs00171409_m1**	0.32124498	0.8318075	3.88177	1.9506061	2.4086914	2.0135813	
**IFNA1-Hs00256882_s1**	10.10777	2.7188652	13.405374	5.995228	0.023557074	0.1658005	
**IFNB1-Hs00277188_s1**	368.30457	0.729463	12.819832	416.32068	0.28179726	453.32138	
**IGF1-Hs00153126_m1**	0.21484588	0.3462328	3.4000778	0.29004368	0.4257926	0.52136433	
**IGF1R-Hs00181385_m1**	2.1515427	1.0385847	0.76515245	1.4102758	0.69486284	0.96284115	
**IL10-Hs00174086_m1**	227.60149			462.6578		557.3716	
**IL12A-Hs00168405_m1**	2.6283464	0.9348713	1.9204093	1.8695375	0.65269613	0.5120319	
**IL8-Hs00174103_m1**	8230.462	192.26549	423.45438	8035.3813	8.580302	8473.69	
**ITGA5-Hs00233743_m1**	0.007016879	0.75174636	2.4165378	0.00529407	0.98316264	0.003165355	
**ITGAV-Hs00233808_m1**	0.13461478	0.5350225	1.4962689	0.23020528	1.0602286	8.66E-05	
**ITGB3-Hs00173978_m1**	3.267746	0.6539382	5.947081	1.5690391	0.98394024	0.5260183	
**MDK-Hs00171064_m1**	3.53E-05	0.09003861	0.49126297	1.14E-05	0.4143062	1.38E-05	
**MMP2-Hs00234422_m1**	0.06501684	0.06270294	0.5394068	0.1453691	0.36595687	0.38970318	
**MMP9-Hs00234579_m1**	114.92867	17.523115					
**NRP1-Hs00826129_m1**	0.19253285	0.40364856	1.4642727	0.42421708	0.84969026	0.59561455	
**PDGFA-Hs00234994_m1**	0.07274194	0.17418255	1.0910153	0.2597842	0.55965406	0.61415255	
**PDGFB-Hs00234042_m1**	5.449627	0.10644139	6.0685534	13.468367	0.10098915	5.118809	
**PDGFRA-Hs00183486_m1**	0.002768794	0.14088596	0.7029478	0.002547684	0.3676712	2.49E-05	
**PDGFRB-Hs00182163_m1**	1.8774015	1.4805268	4.7943697	2.2710285	1.3572775	1.9019116	
**PECAM1-Hs00169777_m1**	1.156894	0.052995175	0.1040989	1.2125528	0.43434724	0.5361058	
**PGF-Hs00182176_m1**	4.7456913	2.105821	13.548447	3.155535	0.5660453	2.1796145	
**PLAU-Hs00170182_m1**	39.47042	1.2292311	6.001818	33.187466	1.4448216	4.6638436	
**PRL-Hs00168730_m1**	0.040201306	0.5173613	1.6739624	0.15236753	0.7588621	0.2555378	
**PTGS1-Hs00168776_m1**	0.17535609	0.6162363	1.9374701	0.615602	0.8635668	0.5756517	
**PTGS2-Hs00153133_m1**	24.201122	2.8172312	10.063408	32.12976	3.3911622	64.61255	
**PTN-Hs00383235_m1**	0.057041302	0.043589298	0.085622855	0.8762439	0.041356523	0.0223033	
**SERPINF1-Hs00171467_m1**	0.12181992	0.08697449	0.9299786	0.25972065	0.33273664	0.3643281	
**SMAD1-Hs00195432_m1**	0.23978347	0.13695763	0.84550935	3.3624253	0.29112205	6.453879	
**SPARC-Hs00277762_m1**	0.25483707	0.55343443	2.253073	0.64047915	1.2052313	1.3904755	
**TGFA-Hs00177401_m1**	40.00497			24.532866	7.7403984	10.875058	
**TGFB1-Hs00171257_m1**	0.6503394	0.44452885	2.2072735	1.0494986	0.9388509	0.7896068	
**TGFB2-Hs00234244_m1**	2.218452	0.6928026	6.918003	2.7012732	1.2703435	1.6092899	
**TGFB3-Hs00234245_m1**	0.75393504	0.29044622	2.5425553	0.6822029	0.43157336	0.5866003	
**TGFBR1-Hs00610319_m1**	0.46907866	0.2305708	1.3967971	0.52385163	0.7293241	0.43068817	
**TGFBR2-Hs00234253_m1**	0.6404681	0.48714763	2.9209936	1.1104643	0.899445	2.5526967	
**TGFBR3-Hs00234257_m1**	0.10452269	0.37017035	2.3282375	0.081267394	0.65085036	0.08022497	
**THBS1-Hs00170236_m1**	1.55E-04	0.259202	0.8546768	4.84E-04	0.30595258	6.04E-05	
**THBS2-Hs00170248_m1**	0.8302957	0.42941177	1.9231178	0.6317223	1.504475	1.0725803	
**THBS3-Hs00200157_m1**	0.1270221	0.002783489	2.011256	0.90325826	1.1349365	0.8794436	
**THBS4-Hs00170261_m1**	0.78752697	0.75311714	1.391818	0.92521054	0.32537183	2.5175822	
**TIE1-Hs00178500_m1**		17.37359		17.331173	16.974987	12.861118	
**TIMP1-Hs00171558_m1**	1.2104434	1.0380837	5.269796	4.445382	0.98651147	0.6582526	
**TIMP2-Hs00234278_m1**	0.29948968	0.39467868	2.031099	0.6166805	0.7404024	0.8702866	
**TNF-Hs00174128_m1**	0.8617454	0.9161413	2.3081508	0.6162013	0.37398857	0.3287523	
**TNFSF15-Hs00353710_s1**	20.48558	3.6079066	18.942228	9.134732	0.49127337	0.74366426	
**VEGF-Hs00173626_m1**	1.56884	1.7600665	5.6470356	1.4351221	0.10570879	0.50813204	
**VEGFB-Hs00173634_m1**	0.26353717	0.59786856	1.6840489	0.31063688	0.8122045	0.28514466	
**VEGFC-Hs00153458_m1**	7599.6533	17.820337		8554.023		6385.3613	
